# Incidence, Risk Factors, and Outcomes of Patients Who Develop Mucosal Barrier Injury–Laboratory Confirmed Bloodstream Infections in the First 100 Days After Allogeneic Hematopoietic Stem Cell Transplant

**DOI:** 10.1001/jamanetworkopen.2019.18668

**Published:** 2020-01-08

**Authors:** Christopher E. Dandoy, Soyoung Kim, Min Chen, Kwang Woo Ahn, Monica I. Ardura, Valerie Brown, Saurabh Chhabra, Miguel Angel Diaz, Christopher Dvorak, Nosha Farhadfar, Aron Flagg, Siddartha Ganguly, Gregory A. Hale, Shahrukh K. Hashmi, Peiman Hematti, Rodrigo Martino, Taiga Nishihori, Roomi Nusrat, Richard F. Olsson, Seth J. Rotz, Anthony D. Sung, Miguel-Angel Perales, Caroline A. Lindemans, Krishna V. Komanduri, Marcie L. Riches

**Affiliations:** 1 Division of Bone Marrow Transplantation and Immune Deficiency, Cincinnati Children’s Hospital Medical Center, Cincinnati, Ohio; 2 Center for International Blood and Marrow Transplant Research, Department of Medicine, Medical College of Wisconsin, Milwaukee; 3 Division of Biostatistics, Institute for Health and Equity, Medical College of Wisconsin, Milwaukee; 4 Division of Infectious Disease, Department of Pediatrics, Nationwide Children’s Hospital, Columbus, Ohio; 5 Division of Pediatric Oncology/Hematology, Department of Pediatrics, Penn State Hershey Children’s Hospital and College of Medicine, Hershey, Pennsylvania; 6 Divsion of Hematology/Oncology, Department of Medicine, Medical College of Wisconsin, Milwaukee; 7 Department of Hematology/Oncology, Hospital Infantil Universitario Nino Jesus, Madrid, Spain; 8 Divsion of Pediatric Allergy, Immunology & Bone Marrow Transplantation, Benioff Children’s Hospital, University of California, San Francisco; 9 Division of Hematology/Oncology, University of Florida College of Medicine, Gainesville; 10 Division of Pediatric Hematology-Oncology, Department of Pediatrics, Yale New Haven Hospital, New Haven, Connecticut; 11 Division of Hematological Malignancy and Cellular Therapeutics, University of Kansas Health System, Kansas City; 12 Department of Hematology/Oncology, Johns Hopkins All Children’s Hospital, St Petersburg, Florida; 13 Department of Internal Medicine, Mayo Clinic, Rochester, Minnesota; 14 Oncology Center, King Faisal Specialist Hospital and Research Center, Riyadh, Saudi Arabia; 15 Division of Hematology/Oncology/Bone Marrow Transplantation, Department of Medicine, University of Wisconsin, Madison; 16 Division of Clinical Hematology, Hospital de la Santa Creu I Sant Pau, Barcelona, Spain; 17 Department of Blood and Marrow Transplantation, H. Lee Moffitt Cancer Center and Research Institute, Tampa, Florida; 18 Department of Medicine, Rutgers Robert Wood Johnson Medical School, New Brunswick, New Jersey; 19 Department of Laboratory Medicine, Karolinska Institutet, Stockholm, Sweden; 20 Centre for Clinical Research Sormland, Uppsala University, Uppsala, Sweden; 21 Department of Pediatric Hematology, Oncology and Blood and Marrow Transplantation, Cleveland Clinic Children’s Hospital, Cleveland, Ohio; 22 Division of Hematologic Malignancies and Cellular Therapy, Department of Medicine, Duke University School of Medicine, Durham, North Carolina; 23 Adult Bone Marrow Transplant Service, Department of Medicine, Memorial Sloan Kettering Cancer Center, New York, New York; 24 Pediatric Blood and Marrow Transplantation Program, University Medical Center Utrecht, Utrecht University, Netherlands; 25 Division of Pediatric Stem Cell Transplantation, Department of Pediatrics, Princess Maxima Center for Pediatric Oncology, Utrecht, the Netherlands; 26 Department of Medicine, University of Miami, Miami, Florida; 27 Division of Hematology/Oncology, The University of North Carolina at Chapel Hill

## Abstract

**Importance:**

Patients undergoing hematopoietic stem cell transplant (HSCT) are at risk for bloodstream infection (BSI) secondary to translocation of bacteria through the injured mucosa, termed *mucosal barrier injury–laboratory confirmed bloodstream infection* (MBI-LCBI), in addition to BSI secondary to indwelling catheters and infection at other sites (BSI-other).

**Objective:**

To determine the incidence, timing, risk factors, and outcomes of patients who develop MBI-LCBI in the first 100 days after HSCT.

**Design, Setting, and Participants:**

A case-cohort retrospective analysis was performed using data from the Center for International Blood and Marrow Transplant Research database on 16 875 consecutive pediatric and adult patients receiving a first allogeneic HSCT from January 1, 2009, to December 31, 2016. Patients were classified into 4 categories: MBI-LCBI (1481 [8.8%]), MBI-LCBI and BSI-other (698 [4.1%]), BSI-other only (2928 [17.4%]), and controls with no BSI (11 768 [69.7%]). Statistical analysis was performed from April 5 to July 17, 2018.

**Main Outcomes and Measures:**

Demographic characteristics and outcomes, including overall survival, chronic graft-vs-host disease, and transplant-related mortality (only for patients with malignant disease), were compared among groups.

**Results:**

Of the 16 875 patients in the study (9737 [57.7%] male; median [range] age, 47 [0.04-82] years) 13 686 (81.1%) underwent HSCT for a malignant neoplasm, and 3189 (18.9%) underwent HSCT for a nonmalignant condition. The cumulative incidence of MBI-LCBI was 13% (99% CI, 12%-13%) by day 100, and the cumulative incidence of BSI-other was 21% (99% CI, 21%-22%) by day 100. Median (range) time from transplant to first MBI-LCBI was 8 (<1 to 98) days vs 29 (<1 to 100) days for BSI-other. Multivariable analysis revealed an increased risk of MBI-LCBI with poor Karnofsky/Lansky performance status (hazard ratio [HR], 1.21 [99% CI, 1.04-1.41]), cord blood grafts (HR, 2.89 [99% CI, 1.97-4.24]), myeloablative conditioning (HR, 1.46 [99% CI, 1.19-1.78]), and posttransplant cyclophosphamide graft-vs-host disease prophylaxis (HR, 1.85 [99% CI, 1.38-2.48]). One-year mortality was significantly higher for patients with MBI-LCBI (HR, 1.81 [99% CI, 1.56-2.12]), BSI-other (HR, 1.81 [99% CI, 1.60-2.06]), and MBI-LCBI plus BSI-other (HR, 2.65 [99% CI, 2.17-3.24]) compared with controls. Infection was more commonly reported as a cause of death for patients with MBI-LCBI (139 of 740 [18.8%]), BSI (251 of 1537 [16.3%]), and MBI-LCBI plus BSI (94 of 435 [21.6%]) than for controls (566 of 4740 [11.9%]).

**Conclusions and Relevance:**

In this cohort study, MBI-LCBI, in addition to any BSIs, were associated with significant morbidity and mortality after HSCT. Further investigation into risk reduction should be a clinical and scientific priority in this patient population.

## Key Points

**Question:**

What outcomes are associated with mucosal barrier injury–laboratory confirmed bloodstream infections in patients who undergo allogeneic hematopoietic stem cell transplant?

**Findings:**

In a case-cohort study of 16 875 pediatric and adult patients who underwent allogeneic hematopoietic stem cell transplant between 2009 and 2016, the cumulative incidence of mucosal barrier injury–laboratory confirmed bloodstream infections was 13% by day 100, with infection occurring a median of 8 days after stem cell transplant. Overall survival was significantly decreased among patients who developed a mucosal barrier injury–laboratory confirmed bloodstream infection.

**Meaning:**

Mucosal barrier injury–laboratory confirmed bloodstream infections are associated with significant morbidity and mortality and, by extension, increased use of health care resources.

## Teaser

This cohort study examines the incidence, timing, risk factors, and outcomes of patients who develop mucosal barrier injury–laboratory confirmed bloodstream infection (MBI-LCBI) in the first 100 days after hematopoietic stem cell transplant (HSCT).

## Introduction

Hematopoietic stem cell transplant (HSCT) is an effective treatment strategy for many malignant neoplasms, marrow failure syndromes, and immune deficiencies in children, adolescents, and adults.^1,2,3,4,5^ Each year, more than 50 000 HSCTs are performed worldwide. Transplant strategies and supportive care have evolved, resulting in improved overall survival (OS)^6^; however, patients who have undergone HSCT remain at high risk for bloodstream infections (BSIs) and associated morbidity and mortality.^5,7,8^

Studies have identified immunocompromised patients, including those who have undergone HSCT, who are at risk of developing BSIs once classified as central line–associated BSIs (CLABSIs) that do not result from contamination of the central venous catheter but instead from other mechanisms such as translocation of bacteria through nonintact mucosa.^9,10^ The Centers for Disease Control and Prevention developed a modification of the CLABSI definition, termed *mucosal barrier injury–laboratory confirmed bloodstream infection* (MBI-LCBI) through literature review and expert opinion.^11,12^ This definition was integrated into National Healthcare Safety Network methods for primary BSI surveillance to classify a subset of BSIs reported as CLABSI that are associated with mucosal barrier injury and not the presence of a central venous catheter.^9^ Unlike CLABSI,^13,14,15^ MBI-LCBIs are not prevented by improved central venous catheter maintenance care.^9,12,16^

A BSI is defined as an MBI-LCBI if it resulted from 1 or more of a group of selected organisms known to be commensals of the oral cavity or gastrointestinal tract and it occurred in a patient with specific signs or symptoms compatible with the presence of mucosal barrier injury, such as gastrointestinal graft-vs-host disease (GVHD) and/or neutropenia.^9,11,12^ To our knowledge, there are few data describing the incidence, risk factors, or outcomes of patients who develop an MBI-LCBI after HSCT. This study aims to determine the incidence, timing, risk factors, and outcomes of patients who develop MBI-LCBI in the first 100 days after HSCT.

## Methods

### Data Source

We analyzed data from the Center for International Blood and Marrow Transplant Research (CIBMTR) registry to compare the outcomes of patients with BSIs. The CIBMTR comprises a voluntary working group of more than 400 transplant centers worldwide that contribute detailed data on allogeneic and autologous HSCTs. The details regarding the CIBMTR and its data collection method are in the eAppendix in the Supplement. This process occurred under the guidance of the CIBMTR via the National Marrow Donor Program Institutional Review Board. Patients provided written informed consent. This study followed the Strengthening the Reporting of Observational Studies in Epidemiology (STROBE) reporting guideline.^17^

### Patients

The study population consisted of all pediatric and adult patients undergoing first allogeneic HSCT reported to the CIBMTR between January 1, 2009, and December 31, 2016, including both malignant and nonmalignant conditions (eFigure 1 in the Supplement). The study included patients receiving umbilical cord blood, bone marrow, or peripheral blood stem cell grafts. To limit center bias, patients were included only from centers in which at least 1 patient with MBI-LCBI was identified, and either 1 control patient or 1 patient with BSI secondary to indwelling catheters and infection at other sites (BSI-other) was present. In addition, we excluded patients from centers reporting no GVHD prophylaxis in more than 15% of patients because this may indicate that other data are incomplete.

### BSI Classification

Centers report infections to the CIBMTR using an organism code, a site code, and the date of the infection. There are no data provided to assess infection prophylaxis, treatment, diagnostic criteria used by the center, or infection severity. Centers are instructed to report clinically significant infections with both online and in-person education regarding appropriate reporting.^18^ Patients were classified into 1 of 4 groups based on BSIs during the first 100 days after HSCT. The first group was the MBI-LCBI cohort, comprising patients who developed at least 1 MBI-LCBI in the first 100 days after transplant (and no BSI-other). Patients in the MBI-LCBI cohort were classified as such if the infection met the following criteria: the organism was a commensal of the oral cavity or gastrointestinal tract, and infection occurred 14 days before or 60 days after stage 3 or 4 gastrointestinal acute GVHD diagnosis or an absolute neutrophil count of more than 500 cells/μL (to convert to 10^9^ cells per liter, multiply by 0.001) was never achieved after HSCT or the infection occurred before or within 3 days of an absolute neutrophil count of 500 cells/μL or less at any time in the first 100 days after HSCT. The second group was the BSI-other group, comprising patients who developed at least 1 fungal or bacterial BSI by 100 days after transplant that did not meet criteria for MBI-LCBI. The third group was the MBI-LCBI and BSI group, comprising patients who developed at least 1 MBI-LCBI and at least 1 BSI-other in the first 100 days after transplant. The fourth group was the control group, comprising recipients of allogeneic HSCT who did not develop a bacterial or fungal BSI documented in the first 100 days.

### Outcomes and Study Definitions

We compared OS in the first year after HSCT between patients in each cohort. The cumulative incidences of MBI-LCBI and BSI-other, with death as the competing risk, were assessed in the first 100 days. We calculated infection density, determined as the number of infections per patient per 100 days, for MBI-LCBI and BSI separately. We computed the frequency of infection as a primary or secondary cause of death within the first year after HSCT as reported by the center. The cumulative incidence function (using relapse or progression as a competing risk) was used to estimate transplant-related mortality (TRM), defined as the time to death without evidence of disease relapse.^19,20,21^ Thus, only patients with malignant disease have a TRM estimate. Furthermore, for patients with malignant disease, we evaluated disease relapse using the cumulative incidence function with death in remission as the competing event.

### Patient-, Disease-, and Transplant-Related Variables

The clinical data of patients were described, including demographic characteristics, disease and therapy characteristics, transplant complications, and outcomes. The following variables were evaluated: sex, age at transplant, diagnosis, donor relationship, HLA match, source of stem cell graft, conditioning intensity,^22^ and neutrophil engraftment. Currently accepted clinical criteria were used for the diagnosis of acute GVHD,^23^ transplant-associated thrombotic microangiopathy,^24,25^ and engraftment syndrome in recipients of allogeneic HSCT.^26^

### Statistical Analysis

Statistical analysis was performed from April 5 to July 17, 2018. Because MBI-LCBI is a time-dependent variable, we used a dynamic landmark study with 3 landmark time points at 30, 60, and 100 days to graphically show the probability of 1-year OS.^27^

Multivariable Cox proportional hazards regression analysis with an examination of the proportional hazards assumption was used to evaluate potential risk factors for MBI-LCBI and for survival. For the Cox proportional hazards regression model for survival, infections and acute GVHD were used as time-dependent variables. If the proportional hazards assumption was violated, the variable was added as a time-dependent covariate. A stepwise selection procedure with a significance level of *P* < .10 was used to identify the final model. Pairwise interactions and center effects were tested.^28^ If center effects were significant, we adjusted them in the final model. Hazard ratios (HRs) and their 99% CIs, using the Wald confidence limit in the final model, were reported. All *P* values were from 2-sided tests and results were deemed statistically significant at *P* = .01.

For the assessment of risk factors for the development of an MBI-LCBI, only the subset of patients with malignant disease was analyzed. The variables examined are shown in eTable 1 in the Supplement.

## Results

### Patient Population

From 2009 to 2016, 22 393 pediatric and adult patients undergoing allogeneic HSCT were reported to the CIBMTR. eFigure 1 in the Supplement depicts the exclusions resulting in the final population of 16 875 patients. For the risk factor analysis for the development of MBI-LCBI, only the subset of 13 686 patients with malignant disease (1.1%) were examined owing to different clinical characteristics and preceding therapies.

Of the 16 875 patients (9737 [57.7%] male; median [range] age, 47 [0.04-82] years), 1481 (8.8%) had at least 1 MBI-LCBI, 2928 (17.4%) developed at least 1 BSI-other, 698 (4.1%) developed both an MBI-LCBI and BSI-other, 3189 (18.9%) underwent HSCT for a nonmalignant condition, and 11 768 (69.7%) did not develop a bacterial or fungal BSI in the first 100 days (control group). The demographic and transplant characteristics of the 4 cohorts of patients are shown in Table 1.

**Table 1.** Characteristics of Patients Who Underwent First Allogeneic Transplants With MBI-LCBI and Without MBI-LCBI by Day 100 After Transplant, Reported to the CIBMTR, 2009-2016

Variable	Patients, No. (%)			
	MBI-LCBI Only	BSI-Other Only	MBI-LCBI + BSI-Other	Control
No. of patients	1481	2928	698	11 768
No. of centers	176	180	151	186
Male sex	863 (58.3)	1715 (58.6)	391 (56.0)	6768 (57.5)
Age, median (range), y	42 (<1-82)	43 (<1-79)	39 (<1-77)	48 (<1-81)
≤20	482 (32.5)	958 (32.7)	221 (31.7)	3030 (25.7)
>20	996 (67.3)	1970 (67.3)	477 (68.3)	8738 (74.3)
HSCT comorbidity index				
0	545 (36.8)	1069 (36.5)	251 (36.0)	4209 (35.8)
1	205 (13.8)	392 (13.4)	93 (13.3)	1614 (13.7)
2	162 (10.9)	353 (12.1)	68 (9.7)	1422 (12.1)
≥3	538 (36.3)	1076 (36.7)	277 (39.7)	4360 (37.0)
Diagnosis				
AML	620 (41.8)	1157 (39.5)	290 (41.5)	4818 (40.9)
ALL	284 (19.2)	480 (16.4)	133 (19.1)	1626 (13.8)
MDS	328 (22.1)	724 (24.7)	146 (20.9)	3260 (27.7)
Severe aplastic anemia	74 (5.0)	129 (4.4)	42 (6.0)	633 (5.4)
Erythrocyte abnormality	59 (4.0)	139 (4.7)	24 (3.4)	649 (5.5)
Immune deficiency	63 (4.3)	149 (5.1)	31 (4.4)	490 (4.2)
Metabolic disorder	31 (2.1)	98 (3.3)	22 (3.2)	165 (1.4)
Histiocytic disorders	22 (1.5)	52 (1.8)	10 (1.4)	127 (1.1)
Graft type				
Bone marrow	313 (21.1)	657 (22.4)	125 (17.9)	2650 (22.5)
Peripheral blood	618 (41.7)	1457 (49.8)	303 (43.4)	7125 (60.5)
Cord blood	550 (37.1)	814 (27.8)	270 (38.7)	1993 (16.9)
HLA match				
Cord blood	550 (37.1)	814 (27.8)	270 (38.7)	1993 (16.9)
HLA-identical siblings	346 (23.4)	677 (23.1)	128 (18.3)	3574 (30.4)
Matched related	8 (0.5)	28 (1.0)	3 (0.4)	125 (1.1)
Mismatched related	78 (5.3)	146 (5.0)	40 (5.7)	537 (4.6)
Related, HLA missing	19 (1.3)	48 (1.6)	12 (1.7)	176 (1.5)
8/8 Unrelated	352 (23.8)	921 (31.5)	174 (24.9)	4185 (35.6)
≤7/8 Unrelated	100 (6.8)	236 (8.1)	62 (8.9)	1141 (9.7)
Unrelated, match missing	28 (1.9)	58 (2.0)	9 (1.3)	280 (2.4)
Conditioning regimen intensity				
Myeloablative	870 (58.7)	1594 (54.4)	419 (60.0)	5740 (48.8)
RIC or NMA	362 (24.4)	767 (26.2)	150 (21.5)	3964 (33.7)
Nonmalignant disease	249 (16.8)	567 (19.4)	129 (18.5)	2064 (17.5)
GVHD prophylaxis				
Ex vivo T-cell depletion	12 (0.8)	33 (1.1)	12 (1.7)	103 (0.9)
CD34 selection	34 (2.3)	42 (1.4)	11 (1.6)	245 (2.1)
Cyclophosphamide	132 (8.9)	200 (6.8)	64 (9.2)	751 (6.4)
TAC or CSA + MMF with or without others	544 (36.7)	1082 (37.0)	286 (41.0)	3480 (29.6)
TAC or CSA + MTX with or without others	559 (37.7)	1189 (40.6)	251 (36.0)	5582 (47.4)
TAC or CSA + others or TAC or CSA alone	185 (12.5)	353 (12.1)	62 (8.9)	1416 (12.0)
Other GVHD prophylaxis	15 (1.0)	39 (1.3)	12 (1.7)	191 (1.6)
Total body irradiation dose				
No total body irradiation	815 (55.0)	1772 (60.5)	383 (54.9)	7637 (64.9)
<1200 cGy	237 (16.0)	501 (17.1)	105 (15.0)	2121 (18.0)
≥1200 cGy	429 (29.0)	655 (22.4)	210 (30.1)	2009 (17.1)
Antithymocyte globulin				
Yes	466 (31.5)	1009 (34.5)	222 (31.8)	3701 (31.4)
Alemtuzumab				
Yes	96 (6.5)	173 (5.9)	39 (5.6)	671 (5.7)
KGF (palifermin) in MAC, TBI ≥1200 cGy (n = 3291)				
No	299 (20.2)	416 (14.2)	147 (21.1)	1101 (9.4)
Yes	43 (2.9)	85 (2.9)	24 (3.4)	295 (2.5)
Missing	87 (5.9)	152 (5.2)	36 (5.2)	606 (5.1)
Year of transplant				
2009	230 (15.5)	589 (20.1)	157 (22.5)	1375 (11.7)
2010	163 (11.0)	336 (11.5)	87 (12.5)	963 (8.2)
2011	100 (6.8)	228 (7.8)	58 (8.3)	720 (6.1)
2012	114 (7.7)	196 (6.7)	47 (6.7)	843 (7.2)
2013	183 (12.4)	356 (12.2)	71 (10.2)	1623 (13.8)
2014	235 (15.9)	412 (14.1)	85 (12.2)	2190 (18.6)
2015	252 (17.0)	444 (15.2)	97 (13.9)	2130 (18.1)
2016	204 (13.8)	367 (12.5)	96 (13.8)	1924 (16.3)
Median follow-up, mo (range)	36 (3-103)	38 (3-102)	47 (3-105)	36 (2-104)

Abbreviations: ALL, acute lymphoblastic leukemia; AML, acute myelogenous leukemia; BSI, bloodstream infection; CSA, cyclosporine; CIBMTR, Center for International Blood and Marrow Transplant Research; GVHD, graft-vs-host disease; HSCT, hematopoietic stem cell transplant; KGF, keratinocyte growth factor; MAC, myeloablative conditioning; MBI-LCBI, mucosal barrier injury–laboratory confirmed bloodstream infection; MDS, myelodysplastic syndromes; MMF, mycophenolate mofetil; MTX, methotrexate; NMA, nonmyeloablative; RIC, reduced intensity conditioning; TAC, tacrolimus.

### Incidence and Timing of BSI After HSCT

**Figure 1.**
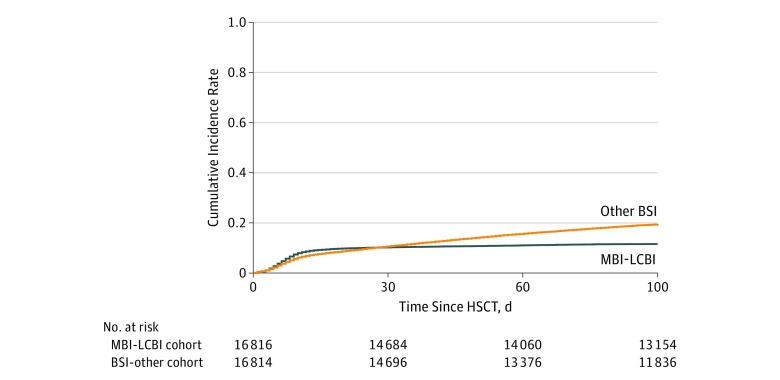
Cumulative Incidence of Bloodstream Infection in the First 100 Days After Allogeneic Hematopoietic Stem Cell Transplantation (HSCT) Patients in the mucosal barrier injury–laboratory confirmed bloodstream infection (MBI-LCBI) plus other bloodstream infection (BSI) cohort are represented in both curves.

The cumulative incidence of MBI-LCBI was 13% (99% CI, 12%-13%) by day 100, whereas the probability of BSI not meeting MBI-LCBI criteria was 21% (99% CI, 21%-22%) by day 100. The median (range) time from transplant to first MBI-LCBI was 8 (<1 to 98) days, MBI-LCBI plus BSI-other was 8 (<1 to 97) days, and BSI-other was 29 (<1 to 100) days. Most cases of MBI-LCBI occurred in the first 2 weeks after HSCT, whereas the incidence of BSI-other continued to increase throughout the first 100 days after HSCT (Figure 1). Most cases of MBI-LCBI met the definition secondary to neutropenia alone (1915 of 2179 [87.9%]), with the remaining 12.1% (264 of 2179) meeting criteria owing to the presence of gastrointestinal GVHD (166 of 2179 [7.6%]) or gastrointestinal GVHD with neutropenia (98 of 2179 [4.5%]). Reported organisms and infection density, accounting for multiple infections, are shown in eTable 2 and eFigure 2 in the Supplement.

### Risk Factors for MBI-LCBI

Table 2 shows the risk factors associated with MBI-LCBI. Multivariable analysis revealed an increased risk of MBI-LCBI in those with a lower Karnofsky/Lansky performance status (score <90) (HR, 1.21 [99% CI, 1.04-1.41]). In addition, myeloablative conditioning (HR, 1.46 [99% CI, 1.19-1.78]), posttransplant cyclophosphamide as GVHD prophylaxis (HR, 1.85 [99% CI, 1.38-2.48]), and receipt of cord blood (HR, 2.89 [99% CI, 1.97-4.24]) were associated with a significant increase in the risk of MBI-LCBI. Preceding GVHD was not examined because it is incorporated in the definition of MBI-LCBI. The results are adjusted for center effects.

**Table 2.** Multivariate Analysis of Variables Associated With MBI-LCBI

Variable	No. of Patients	Hazard Ratio (99% CI)	*P* Value	Overall *P* Value
Karnofsky/Lanksy performance status				
≥90	8568	1.00 [Reference]	NA	.006
<90	5095	1.21 (1.04-1.41)	.002	
Missing	202	1.02 (0.59-1.78)	.92	
Conditioning regimen intensity				
RIC or NMA	5243	1.00 [Reference]	NA	<.001
Myeloablative	8622	1.46 (1.19-1.78)	<.001	
GVHD prophylaxis				
TAC or CSA + MTX with or without others	6474	1.00 [Reference]	NA	<.001
TAC or CSA + MMF with or without others	4453	0.84 (0.66-1.07)	.06	
TAC or CSA with or without others	1442	0.81 (0.58-1.12)	.09	
CD34 selection or ex vivo TCD	314	1.34 (0.78-2.30)	.17	
Cyclophosphamide	1009	1.85 (1.38-2.48)	<.001	
Other GVHD prophylaxis	173	0.65 (0.34-1.23)	.08	
Graft type and donor type				
Matched related BM	644	1.00 [Reference]	NA	<.001
Mismatched related BM	251	1.07 (0.61-1.89)	.75	
8/8 Unrelated BM	941	1.08 (0.71-1.64)	.65	
Mismatched unrelated BM	221	1.53 (0.81-2.88)	.09	
Matched related PBSC	3275	0.91 (0.65-1.27)	.47	
Mismatched related PBSC	392	1.15 (0.63-2.10)	.54	
8/8 Unrelated PBSC	4049	0.87 (0.61-1.26)	.34	
Mismatched unrelated PBSC	861	1.11 (0.72-1.71)	.53	
Cord blood	2731	2.89 (1.97-4.24)	<.001	
Missing	500	0.86 (0.50-1.50)	.49	

Abbreviations: BM, bone marrow; CSA, cyclosporine; GVHD, graft vs host disease; MBI-LCBI, mucosal barrier injury–laboratory confirmed bloodstream infections; MMF, mycophenolate mofetil; MTX, methotrexate; NA, not applicable; NMA, nonmyeloablative; PBSC, peripheral blood stem cell; RIC, reduced intensity conditioning; TAC, tacrolimus; TCD, T-cell depleted.

### Outcomes

**Figure 2.**
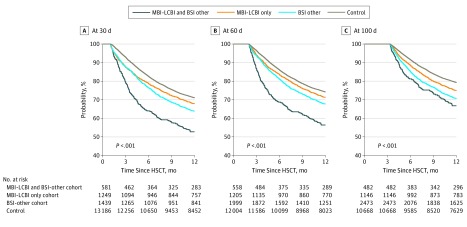
Overall Survival at 1 Year After Allogeneic Hematopoietic Stem Cell Transplant (HSCT) A, Occurrence of infection of interest at 30 days. B, Occurrence of infection of interest at 60 days. C, Occurrence of infection of interest at 100 days. Mucosal barrier injury–laboratory confirmed bloodstream infection (MBI-LCBI) cohort includes those with at least 1 MBI-LCBI, bloodstream infection (BSI)-other cohorts include those with at least 1 BSI that is not classified as an MBI-LCBI, MBI-LCBI and BSI-other group includes those with at least 1 MBI-LCBI and BSI-other, and the control group includes those who underwent allogeneic transplant and did not have any BSI documented in the first 100 days after transplant.

Overall mortality was higher for patients with MBI-LCBI only (HR, 1.81 [99% CI, 1.56-2.12]), BSI only (HR, 1.81 [99% CI, 1.60-2.06]), and MBI-LCBI plus BSI-other (HR, 2.65 [99% CI, 2.17-3.24]) compared with controls (Table 3). A center effect was noted, and the results were adjusted. Figure 2 depicts the OS curves as a series of dynamic landmark analyses examining the outcome of infection by day 30, day 60, and day 100. For patients alive at day 100, the 1-year survival was inferior for patients with MBI-LCBI (n = 1146 [75.1%]; 99% CI, 71.6%-78.3%), BSI only (n = 2473 [70.8%]; 99% CI, 68.3%-73.1%), or MBI-LCBI plus BSI-other (n = 482 [66.8%]; 99% CI, 61.1%-72.2%) compared with controls (n = 10 668 [79.3%]; 99% CI, 78.2%-80.3%; *P* < .001). Additional factors associated with survival are shown in Table 3.

**Table 3.** Multivariate Analysis of Risk Factors For Mortality in Patients Receiving Allogeneic Hematopoietic Stem Cell Transplant^a^

Variable	No. of Patients	Hazard Ratio of Death (99% CI)	*P* Value	Overall *P* Value
Main outcome				
Control	11 768	1.00 [Reference]	NA	<.001
MBI-LCBI only	1481	1.81 (1.56-2.12)	<.001	
Other BSI only	2928	1.81 (1.60-2.06)	<.001	
MBI-LCBI and other BSI	698	2.65 (2.17-3.24)	<.001	
Age at transplant, y				
≤20	4691	1.00 [Reference]		<.001
21-40	2658	1.17 (0.98-1.40)	.02	
41-60	4921	1.51 (1.26-1.80)	<.001	
≥61	4605	1.76 (1.42-2.19)	<.001	
Karnofsky/Lansky performance status				
≥90	10 835	1.00 [Reference]		<.001
<90	5766	1.35 (1.20-1.51)	<.001	
Missing	274	1.37 (0.90-2.09)	.06	
HCT-CI				
0	6074	1.00 [Reference]		<.001
1-2	4309	1.07 (0.95-1.21)	.16	
≥3	6251	1.38 (1.19-1.61)	<.001	
Missing	241	0.79 (0.50-1.24)	.18	
Disease				
Nonmalignant	3009	1.00 [Reference]		<.001
AML	6885	1.56 (1.24-1.96)	<.001	
ALL	2523	1.33 (1.04-1.69)	.003	
MDS	4458	1.55 (1.22-1.96)	<.001	
GVHD prophylaxis				
TAC or CSA + MTX with or without others	7581	1.00 [Reference]		<.001
TAC or CSA + MMF with or without others	5392	1.20 (1.05-1.38)	<.001	
TAC or CSA with or without others (except MTX or MMF)	2006	1.13 (0.94-1.36)	.10	
CD34 selection or ex vivo TCD	492	1.15 (0.74-1.80)	.41	
Cyclophosphamide	1147	1.16 (0.92-1.48)	.10	
Other GVHD prophylaxis	257	1.43 (1.08-1.91)	.001	
ATG or campath				
No	10 510	1.00 [Reference]		.003
Yes	6365	1.17 (1.02-1.33)		
Year of transplant				
2009-2011	5006	1.00 [Reference]		.005
2012-2014	6355	0.88 (0.78-0.99)	.006	
2015-2016	5514	0.83 (0.71-0.97)	.002	
Graft type and donor type				
Matched related bone marrow	1395	1.00 [Reference]		<.001
Mismatched related bone marrow	315	1.25 (0.90-1.73)	.08	
8/8 Unrelated bone marrow	1460	1.21 (0.90-1.62)	.09	
Mismatched unrelated bone marrow	374	1.56 (1.09-2.24)	.001	
Matched related peripheral blood	3494	1.14 (0.81-1.60)	.33	
Mismatched related peripheral blood	486	1.39 (0.83-2.35)	.10	
8/8 Unrelated peripheral blood	4172	1.07 (0.78-1.48)	.57	
Mismatched unrelated peripheral blood	922	1.44 (1.00-2.08)	.01	
Cord blood	3627	1.54 (1.12-2.11)	<.001	
Missing	630	1.46 (0.99-2.14)	.01	
Acute GVHD grade 2-4				
No	10536	1.00 [Reference]		<.001
Yes	6339	1.56 (1.36-1.79)		

Abbreviations: ALL, acute lymphoblastic leukemia; AML, acute myeloid leukemia; ATG, antithymocyte globulin; BSI, bloodstream infection; CSA, cyclosporine; GVHD, graft-vs-host disease; HCT-CI, hematopoietic cell transplant comorbidity index; MBI-LCBI, mucosal barrier injury–laboratory confirmed bloodstream infections; MDS, myelodysplastic syndrome; MMF, mycophenolate mofetil; MTX, methotrexate; TAC, tacrolimus; TCD, T-cell depleted.

^a^ There were 16 875 pediatric and adult patients.

One-year TRM (nonrelapse mortality) among patients with malignant disease increased for patients with any BSI. The increased risk was similar for patients with MBI-LCBI (HR, 2.34 [99% CI, 1.95-2.80]) or BSI-other (HR, 2.12 [99% CI, 1.78-2.52]) but further worsened for patients with MBI-LCBI plus BSI-other (HR, 3.93 [99% CI, 3.10-4.97]) compared with controls. There was no association of any BSI with the development of chronic GVHD. Additional factors associated with TRM and chronic GVHD are listed in eTable 3 in the Supplement.

Infection was reported as the primary cause of death more often for patients with MBI-LCBI (139 of 740 [18.8%]), BSI only (251 of 1537 [16.3%]), and MBI-LCBI plus BSI (94 of 435 [21.6%]) than for controls (566 of 4740 [11.9%]) (*P* < .001). In addition, infection as an associated secondary cause of death was higher in patients with MBI-LCBI (158 of 740 [21.4%]), BSI only (343 of 1537 [22.3%]), and MBI-LCBI plus BSI (116 of 435 [26.7%]) than in with controls (739 of 4740 [15.6%]).

## Discussion

In this large study, we report a high incidence of MBI-LCBI in recipients of allogeneic HSCT. Moreover, MBI-LCBI, similar to BSI-other, was associated with decreased OS as well as increased TRM. Furthermore, infection was more commonly reported as the primary or secondary cause of death for patients with MBI-LCBI or BSI. These data indicate that a reduction in BSI should be a key target for quality-improvement work to reduce mortality, morbidity, and consumption of health care resources.

Multivariable analysis of risk factors identified an increased risk of MBI-LCBI in patients with poor performance status, cord blood grafts, myeloablative conditioning, and posttransplant cyclophosphamide GVHD prophylaxis. Delayed engraftment is seen with umbilical cord blood grafts, increasing the time patients are at risk for MBI-LCBI. These data support current efforts to use umbilical cord blood graft expansion to reduce the duration of neutropenia. The increased risk seen with myeloablative conditioning likely reflects greater mucosal barrier injury and provides another focus for quality-improvement efforts. The increase in MBI-LCBI in patients receiving posttransplant cyclophosphamide may be associated with increased mucositis leading to susceptibility to translocation of bacteria into the bloodstream.

Reported evidence over the last decade shows that major progress has been made in preventing CLABSIs.^13,29,30,31^ However, to our knowledge, there are few data describing the mechanisms to decrease MBI-LCBIs. One of the original incentives for defining MBI-LCBI was to separate infections that could be reduced by attention to central venous catheter care from those that could not. In support of this definition, data demonstrate no change in MBI-LCBI rates with CLABSI prevention standard compliance, while the interventions were associated with CLABSIs.^9,12,16^ Although MBI-LCBI may not be amenable to central venous catheter care interventions, our data show that these infections are still associated with significant patient morbidity and mortality and that these infections are prevalent in this population.^32,33^ Mucosal barrier injury–laboratory confirmed bloodstream infections are associated with significant health care resource use. A single-center retrospective analysis demonstrated that 40% of patients with an MBI-LCBI required central venous catheter removal, 46% of patients developed septic shock at the time of blood culture, 23% of patients were transferred to the intensive care unit within 48 hours of infection and that all-cause mortality within 10 days was 9%.^34^

The National Healthcare Safety Network (NHSN) created the MBI-LCBI definition in 2013 to enable surveillance staff in hospitals to identify and report BSIs in oncology patients and those undergoing HSCT that likely were the result of mucosal barrier injury and therefore not preventable through recommended central line insertion and maintenance practices. There are limitations to the National Healthcare Safety Network’s MBI-LCBI classification scheme. The National Healthcare Safety Network list is likely not inclusive of all organisms that may cause BSI, owing to translocation across compromised oral or gastrointestinal mucosa.^32^ To support this, Tamburini et al^35^ demonstrated that organisms not classically thought to originate in the gut may develop a reservoir, leading to bacterial translocation (eg, *Pseudomonas aeruginosa* strains in the gut microbiome of a patient undergoing HSCT and in a subsequent BSI from the same individual). In addition, an absolute neutrophil count of greater than 500 cells/μL (a key part of the definition), is not necessarily associated with an intact mucosa.

### Limitations and Strengths

Our study has limitations inherent to the registry database. First, our classification of MBI-LCBI is limited to the organisms in the National Healthcare Safety Network and correlated with the dates of neutrophil engraftment or subsequent decrease in neutrophil count and the onset of stage 3 or stage 4 acute GVHD as reported by centers. Consequently, there may be patients in the BSI-other group that actually had MBI-LCBI and vice versa. However, given the large number of patients in this study and the rigor used in data verification for engraftment and acute GVHD by the CIBMTR, this possibility is unlikely to have a significant association with our results. Second, there are no data captured on antibiotic prophylaxis or treatment, which may have varied considerably across centers and over time. Our analysis attempted to account for these variations by limiting centers to those with at least 1 patient with MBI-LCBI, with patients in the control and/or BSI-other category as centers apply antimicrobial prophylaxis and treatment in a standard manner across patients. Third, the degree of mucosal injury is a key factor for translocation of bacteria in the bloodstream; however, the severity of mucositis is not reported. Our finding of increased risk for recipients of myeloablative preparative regimens and those receiving posttransplant cyclophosphamide supports a role for the severity of mucositis. In contrast, use of palifermin, intended to decrease mucositis,^36,37,38^ was different across the 4 cohorts, with a slightly lower frequency in the control cohort. However, the small numbers of patients receiving palifermin in our cohort limited the examination in multivariable analysis. Fourth, the true association of MBI-LCBI with chronic GVHD may be underestimated owing to the time frame of the assessments in this cohort.

Our study has several strengths, including a robust sample size from 186 centers from diverse geographical locations and reflecting current transplant practices. In addition, to our knowledge, this is the first large-scale study to evaluate MBI-LCBI. The inclusion of multiple centers provides a diverse population of all ages, stem cell sources, and transplant types and minimizes overreporting or underreporting biases inherent in single-center studies. Uniform definitions were used for data collection stipulated by the CIBMTR, and long-term follow-up is ensured.

## Conclusions

We found that MBI-LCBI, particularly in combination with another BSI, is negatively associated with post-HSCT outcomes and presents a burden to our health care system. Reduction in MBI-LCBI will require a better understanding of its mechanisms and risk factors, and our data contribute to the knowledge needed to make important progress.

## Notes

Supplement. **eAppendix.** Methods **eTable 1.** Variables Examined in the Cox Proportional Hazards Models for Overall Survival, Transplant Related Mortality, Chronic GVHD, and Risk Factor Analysis for Development of MBI-LCBI **eTable 2.** Organisms Identified as Blood Stream Infections in the MBI-LCBI Only, BSI-Other Only, and the MBI-LCBI+BSI-Other Categories **eTable 3.** Outcomes of Patients Included in the Analysis **eFigure 1.** CONSORT Diagram **eFigure 2.** Infection Density Examines the Number of Infections per Days at Risk During the First 100 Days
