# Supplementary material for: Incidence, Risk Factors, and Outcomes of Patients Who Develop Mucosal Barrier Injury–Laboratory Confirmed Bloodstream Infections in the First 100 Days After Allogeneic Hematopoietic Stem Cell Transplant
Source: JAMA Netw Open. 2020 Jan 8;3(1):e1918668. doi: 10.1001/jamanetworkopen.2019.18668 (PMC6991246; doi:10.1001/jamanetworkopen.2019.18668)
Supplement: Supplement. — eAppendix. Methods eTable 1. Variables Examined in the Cox Proportional Hazards Models for Overall Survival, Transplant Related Mortality, Chronic GVHD, and Risk Factor Analysis for Development of MBI-LCBI eTable 2. Organisms Identified as Blood Stream Infections in the MBI-LCBI Only, BSI-Other Only, and the MBI-LCBI+BSI-Other Categories eTable 3. Outcomes of Patients Included in the Analysis eFigure 1. CONSORT Diagram eFigure 2. Infection Density Examines the Number of Infections per Days at Risk During the First 100 Days [file jamanetwopen-3-e1918668-s001.pdf]

Supplementary Online Content

Dandoy CE, Kim S, Chen M, et al. Incidence, risk factors, and outcomes of patients who develop mucosal barrier injury–laboratory confirmed bloodstream infections in the first 100 days after allogeneic hematopoietic stem cell transplant. *JAMA Netw Open.* 2020;3(1):e1918668. doi:10.1001/jamanetworkopen.2019.18668

eAppendix. Methods
eTable 1. Variables Examined in the Cox Proportional Hazards Models for Overall Survival, Transplant Related Mortality, Chronic GVHD, and Risk Factor Analysis for Development of MBI-LCBI
eTable 2. Organisms Identified as Blood Stream Infections in the MBI-LCBI Only, BSI-Other Only, and the MBI-LCBI+BSI-Other Categories
eTable 3. Outcomes of Patients Included in the Analysis
eFigure 1. CONSORT Diagram
eFigure 2. Infection Density Examines the Number of Infections per Days at Risk During the First 100 Days

This supplementary material has been provided by the authors to give readers additional information about their work.

© 2020 Dandoy CE et al. *JAMA Network Open.*

eAppendix. Methods

Data Source: CIBMTR is a research collaboration between the National Marrow Donor Program®/Be The Match® and the Medical College of Wisconsin. It comprises a voluntary working group of more than 400 transplant centers worldwide that contribute detailed data on allogeneic and autologous HSCT. Participating centers are required to report all transplants consecutively; compliance is monitored by on-site audits, and patients are followed longitudinally. Computerized checks for discrepancies, physicians' review of submitted data, and on-site audits of participating centers ensure data quality. Studies conducted by the CIBMTR are performed in compliance with all applicable federal regulations pertaining to the protection of human research participants. The CIBMTR collects an internationally accepted standard data-set that contains a number of key variables for all consecutive transplant recipients. Data are collected pre-transplant, 100 days and six months post-transplant, annually until year six post-transplant and biannually thereafter until death. Infection data are reported on a subset of patients selected algorithmically for detailed research data. The algorithm is a weighted-randomization selection algorithm in which CIBMTR selects patients for more detailed comprehensive report forms (CRF). The algorithm randomly selects an epidemiologic sample of recipients for whom a CRF is to be requested. The algorithm includes, but is not limited to, type of HCT, age of the recipient, disease, etc. It gives higher weights to patients receiving HCT for rare indications, to very young and very old patients, and novel treatment approaches. It aims to provide representative, adequately sized subsets of patients for studies requiring detailed data. The algorithm is reviewed every 6 – 12 months to assess the burden of data submission for centers. Approximately 75% of CIBMTR centers provide CRF-level data and account for more than 90% of the cases submitted to CIBMTR annually. The algorithm is determined upon baseline characteristics and not upon outcomes or complications such as

© 2020 Dandoy CE et al. *JAMA Network Open.*

infections. However, data on infectious complications are only reported on the CRF and only research data patients are included in this analysis.

Statistical Analysis: Variables examined in the multivariable analyses are shown in Supplemental Table 1.

Results

Organisms: Organisms identified are shown in Supplemental Table 2. For MBI-LCBI, appropriate organisms in the context of neutropenia and/or stage 3-4 GI GVHD are necessary. The organisms for MBI-LCBI include *Candida spp.*, *Enterococcus spp*., *Viridans Streptococci,* Enterobacteriaceae, and anaerobes.

Infection density: Infection density accounts for multiple infections during the first 100 days. Because patients may die prior to day 100, this rate is normalized by patient days at risk. This was examined separately for BSI-Other and for MBI-LCBI infections. For both BSI and MBI-LCBI, patients had a rate of more than 1 infection during the first 100 days with the highest rate for patients in the MBI-LCBI cohort experiencing 1.54 BSI-other infections per patient in the first 100 days post-transplant. Additionally, patients experiencing an MBI-LCBI had a rate of 1.29 – 1.39 MBI-LCBI infections per patient in the first 100 days. These data are shown in Supplemental Figure 1.

Outcomes: Supplemental Table 3 shows the independent factors affecting the risk of transplant-related mortality and chronic GVHD.

© 2020 Dandoy CE et al. *JAMA Network Open.*

eTable 1. Variables Examined in the Cox Proportional Hazards Models for Overall Survival, Transplant Related Mortality, Chronic GVHD, and Risk Factor Analysis for Development of MBI-LCBI

Multivariable analysis for Overall Survival (OS), Transplant Related Mortality (TRM), and Chronic GVHD

- MBI-LCBI vs MBI-LCBI+BSI-other vs BSI-other vs Control (ref) [*main effect variable]*
- Age: ≤ 20 (ref) vs 21-40 vs 41- 60 vs ≥61
- KPS: <90 vs ≥ 90 (ref)
- HCT-CI: 0 (ref) vs 1 – 2 vs ≥ 3
- Graft type+donor type (composite variable): Matched related BM (ref) vs Mismatched related BM vs 8/8 unrelated BM vs Mismatched unrelated (7/8 + 6/8) BM vs Matched related PBSC vs Mismatched related PBSC vs 8/8 unrelated PBSC vs Mismatched unrelated (7/8 + 6/8) PBSC vs Cord Blood vs missing
- Conditioning intensity: Malignant Disease NMA/RIC no TBI (ref) vs Malignant disease MAC + TBI vs Malignant Disease MAC no TBI vs Malignant Disease NMA/RIC + TBI vs Non-malignant disease conditioning + TBI vs Non-Malignant Disease conditioning no TBI
- GVHD Prophylaxis: TAC/CSA + MTX (ref) ± Others vs TAC/CSA + MMF ± Others vs TAC/CSA ± Others vs CD34 selection/ex vivo TCD vs PTCy vs Others
- ATG/CAMPATH: Yes vs No (ref)
- Year of HCT: 2009 – 2011 (ref) vs 2012 – 2014 vs 2015 – 2016
- aGVHD grade 2-4: Yes vs No (ref)

Multivariable Analysis for Risk Factors for MBI-LCBI (AML/ALL/MDS patients only)

- Age: ≤ 20 (ref) vs 21-40 vs 41- 60 vs ≥61
- Disease: AML (ref) vs ALL vs MDS
- KPS: <90 vs ≥ 90 (ref)
- Graft type+donor type (composite variable): Matched related BM (ref) vs Mismatched related BM vs 8/8 unrelated BM vs Mismatched unrelated (7/8 + 6/8) BM vs Matched related PBSC vs Mismatched related PBSC vs 8/8 unrelated PBSC vs Mismatched unrelated (7/8 + 6/8) PBSC vs Cord Blood vs missing
- Conditioning intensity: RIC/NMA(ref) vs Myeloablative
- GVHD Prophylaxis: TAC/CSA + MTX (ref) ± Others vs TAC/CSA + MMF ± Others vs TAC/CSA ± Others vs CD34 selection/ex vivo TCD vs PTCy vs Others
- TBI: Yes vs No (ref)
- ATG/CAMPATH: Yes vs No (ref)
- Prophylactic antibiotics: Yes vs No (ref)

Abbreviations: ALL=acute lymphoblastic leukemia; AML=acute myelogenous leukemia; ATG=Anti-thymocyte globulin; CSA=cyclosporine; GVHD=graft versus host disease; HC-CT=hematopoietic cell transplant co-morbidity index; MAC=myeloablative conditioning; MBI-LCBI=mucosal barrier injury laboratory confirmed bloodstream infections; MDS =myelodysplastic syndromes MMF=mycophenolate mofetil; MTX=methotrexate; NMA=non-myeloablative; RIC=reduced intensity conditioning; TAC=tacrolimus; TBI = total body irradiation

© 2020 Dandoy CE et al. *JAMA Network Open.*

eTable 2. Organisms Identified as Blood Stream Infections in the MBI-LCBI Only, BSI-Other Only, and the MBI-LCBI+BSI-Other Categories

Organisms by category are not mutually exclusive.

**Organism categories**	**MBI-LCBI Only N (%)**	**BSI-Other Only N (%)**	**MBI-LCBI + BSI-Other N (%)**
Candida spp	73 (5)	38 (1)	58 (8)
Enterococcus spp	355 (24)	183 (6)	261 (37)
Strep viridans	410 (28)	88 (3)	183 (26)
Enterobacteriaceae	633 (43)	342 (12)	270 (39)
Anaerobes	155 (10)	106 (4)	113 (16)
Mycobacterium spp	NA	15 (<1)	1 (<1)
GNR, Non-Enterobacteriaceae	NA	389 (13)	100 (14)
Staphyloccocus spp	NA	1758 (60)	442 (63)
Strep pneumoniae	NA	29 (<1)	5 (<1)
Other bacteria	NA	206 (7)	61 (9)
Yeast other than *Candida spp*	NA	14 (<1)	9 (1)
Aspergillus spp	NA	79 (3)	25 (4)
Mold other than *Aspergillus spp*	NA	6 (<1)	3 (<1)
Fungus NOS	NA	40 (1)	16 (2)

© 2020 Dandoy CE et al. *JAMA Network Open.*

eTable 3. Outcomes

			99% CI	99% CI		
**Variables**	**N**	**RR of death**	**Lower Limit**	**Upper Limit**	**p-value**	**overall p-value**
**Transplant Related Mortality (AML/ALL/MDS patients only)**						
**Main effect**						<0.001
Control	9597	1.00				
MBI-LCBI only	1206	2.34	1.95	2.80	<0.001	
BSI-other only	2323	2.12	1.78	2.52	<0.001	
MBI-LCBI and other BSI	560	3.93	3.10	4.97	<0.001	
**Age at transplant, years**						<0.001
<=20	2206	1.00				
21-40	2227	1.42	1.11	1.82	<0.001	
41-60	4739	1.83	1.40	2.40	<0.001	
>=61	4514	2.32	1.68	3.21	<0.001	
**Karnofsky performance Status**						<.0001
>=90	8337	1.00				
<90	4982	1.33	1.14	1.54	<0.001	
Missing	199	1.44	0.83	2.52	0.09	
**HCT-CI**						<0.001
0	4079	1.00				
1 – 2	3663	1.01	0.84	1.21	0.93	
3+	5632	1.34	1.10	1.65	<0.001	
Missing	144	0.66	0.26	1.63	0.23	
**Disease**						<0.001
AML	6763	1.00				
ALL	2464	1.19	1.00	1.41	0.01	
MDS	4291	1.51	1.34	1.69	<0.001	
**Conditioning Intensity**						0.008
RIC/NMA no TBI	3405	1.00				
MAC + TBI	3799	0.97	0.74	1.29	0.81	
MAC no TBI	4701	1.08	0.88	1.32	0.36	
RIC/NMA + TBI	1781	0.76	0.61	0.96	0.003	
**GVHD prophylaxis**						0.002
TAC/CSA + MTX +/- others	6338	1.00				
TAC/CSA + MMF +/- others	4328	1.33	1.10	1.61	<0.001	
TAC/CSA +/- others (except MTX, MMF)	1395	1.15	0.87	1.53	0.20	
CD34 selection/ex vivo TCD	308	1.17	0.69	1.97	0.43	
Cyclophosphamide	983	1.20	0.87	1.65	0.149	
Other GVHD prophylaxis	166	1.54	1.00	2.36	0.01	
**ATG or Campath**						0.005
No	9658	1.00				
Yes	3860	1.24	1.02	1.50		

© 2020 Dandoy CE et al. *JAMA Network Open.*

**Graft type & Donor type**						
Matched related bone marrow	635	1.00				<0.001
Mismatched related bone marrow	249	1.10	0.60	2.02	0.69	
8/8 unrelated bone marrow	920	1.08	0.61	1.89	0.74	
Mismatched unrelated bone marrow	220	2.10	1.09	4.03	0.004	
Matched related peripheral blood	3246	0.95	0.56	1.62	0.81	
Mismatched related peripheral blood	389	1.48	0.72	3.06	0.16	
8/8 unrelated peripheral blood	4015	0.98	0.58	1.66	0.94	
Mismatched unrelated peripheral blood	855	1.42	0.80	2.53	0.11	
Cord blood	2664	1.67	0.96	2.92	0.02	
Missing	493	1.41	0.77	2.58	0.15	
**Acute GVHD grade 2-4**						
No	8239	1.00				<0.001
Yes	5357	2.09	1.76	2.48		
**Chronic GVHD**						
**Main effect**						
Control	11620	1.00				0.11
MBI-LCBI only	1470	1.02	0.87	1.18	0.78	
Other BSI only	2886	1.05	0.95	1.15	0.20	
MBI-LCBI and other BSI	694	0.85	0.67	1.09	0.09	
**GVHD prophylaxis**						
TAC/CSA + MTX +/- others	7497	1.00				<0.001
TAC/CSA + MMF +/- others	5316	1.10	0.91	1.32	0.19	
TAC/CSA +/- others (except MTX, MMF)	1990	1.06	0.84	1.34	0.50	
CD34 selection/ex vivo TCD	485	0.37	0.24	0.59	<0.001	
Cyclophosphamide	1130	0.80	0.63	1.02	0.02	
Other GVHD prophylaxis	252	0.67	0.42	1.07	0.03	
**ATG or Campath**						
No	10355	1.00				<0.001
Yes	6315	0.66	0.57	0.77	<0.001	
**Year of transplant**						
2009-2011	4949	1.00				<0.001
2012-2014	6270	0.82	0.73	0.93	<0.001	
2015-2016	5451	0.74	0.62	0.89	<0.001	
**Graft type & Donor type**						
Bone Marrow and Matched related	1385	1.00				<0.001
Bone Marrow and Mismatched related	313	1.07	0.65	1.79	0.72	
Bone Marrow and 8/8 unrelated	1450	1.73	1.28	2.33	<0.001	

© 2020 Dandoy CE et al. *JAMA Network Open.*

Bone Marrow and Mismatched unrelated	371	2.07	1.42	3.01	<0.001	
Peripheral blood and Matched related	3441	2.44	1.87	3.20	<0.001	
Peripheral blood and Mismatched related	480	2.53	1.68	3.81	<0.001	
Peripheral blood and 8/8 unrelated	4107	2.69	2.03	3.57	<0.001	
Peripheral blood and Mismatched unrelated	913	2.86	2.07	3.95	<0.001	
Cord blood	3587	1.36	1.01	1.85	0.009	
missing	623	2.15	1.51	3.06	<0.001	
**aGVHD grade 2-4**						
No	10340	1.00				<0.001
Yes	6330	1.50	1.35	1.67	<0.001	

© 2020 Dandoy CE et al. *JAMA Network Open.*

eFigure 1. CONSORT Diagram

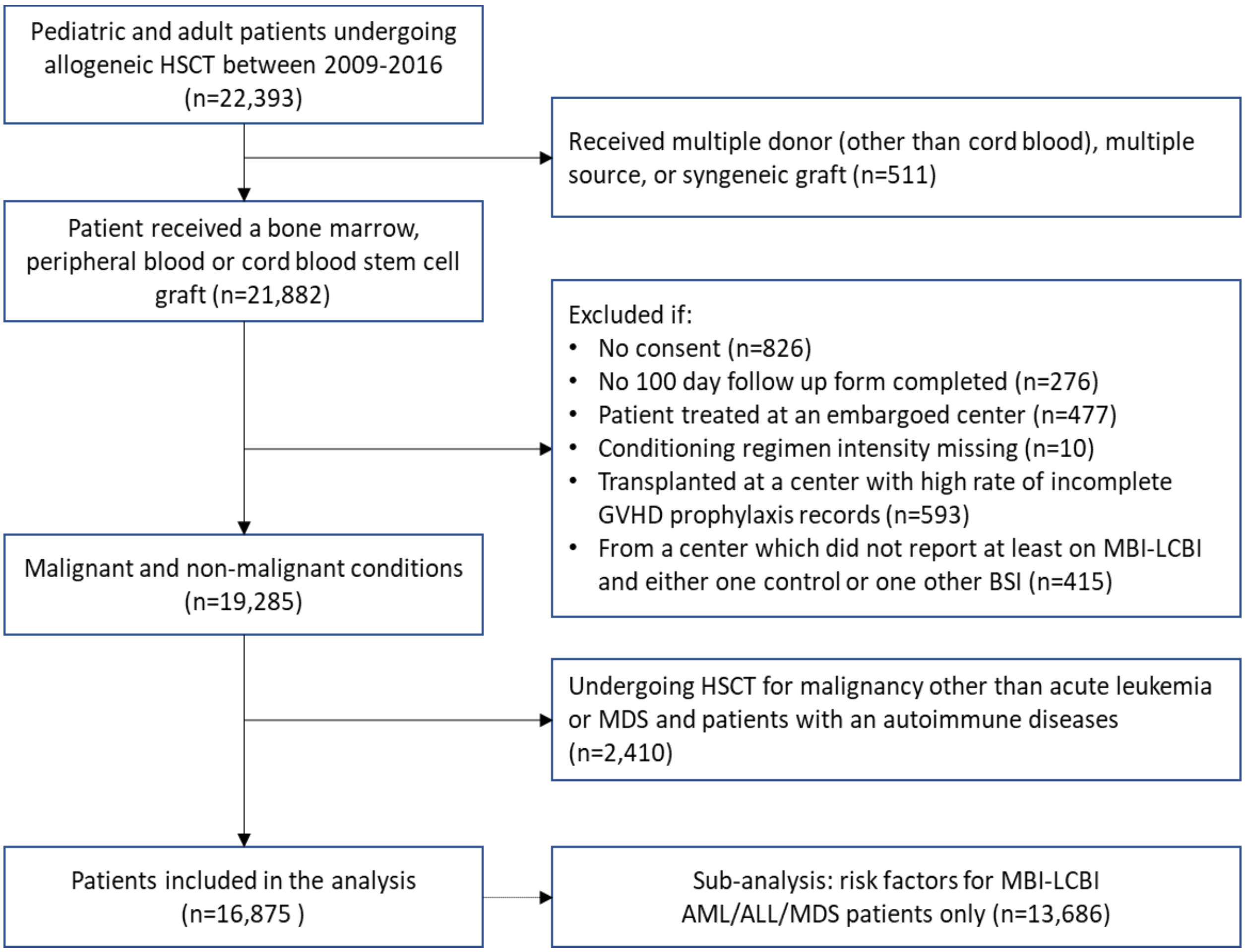

© 2020 Dandoy CE et al. *JAMA Network Open.*

eFigure 2. Infection Density Examines the Number of Infections per Days at Risk During the First 100 Days

The figure shows the rate of any BSI by infection cohort [MBI-BSI, BSI-Other, and MBI-LCBI+BSI-Other]. For BSI only, the rate is 0 for MBI-LCBI group as this cohort and no events of BSI-other only. Similarly, the BSI-other group has 0 MBI-LCBI infections. These data show that for a patient alive for a full 100 days, there are 1.43 BSI-Other infections/patient by day 100 and this increases to 1.54 BSI-Other infections/patient by day 100 if the patient also had an MBI-LCBI.

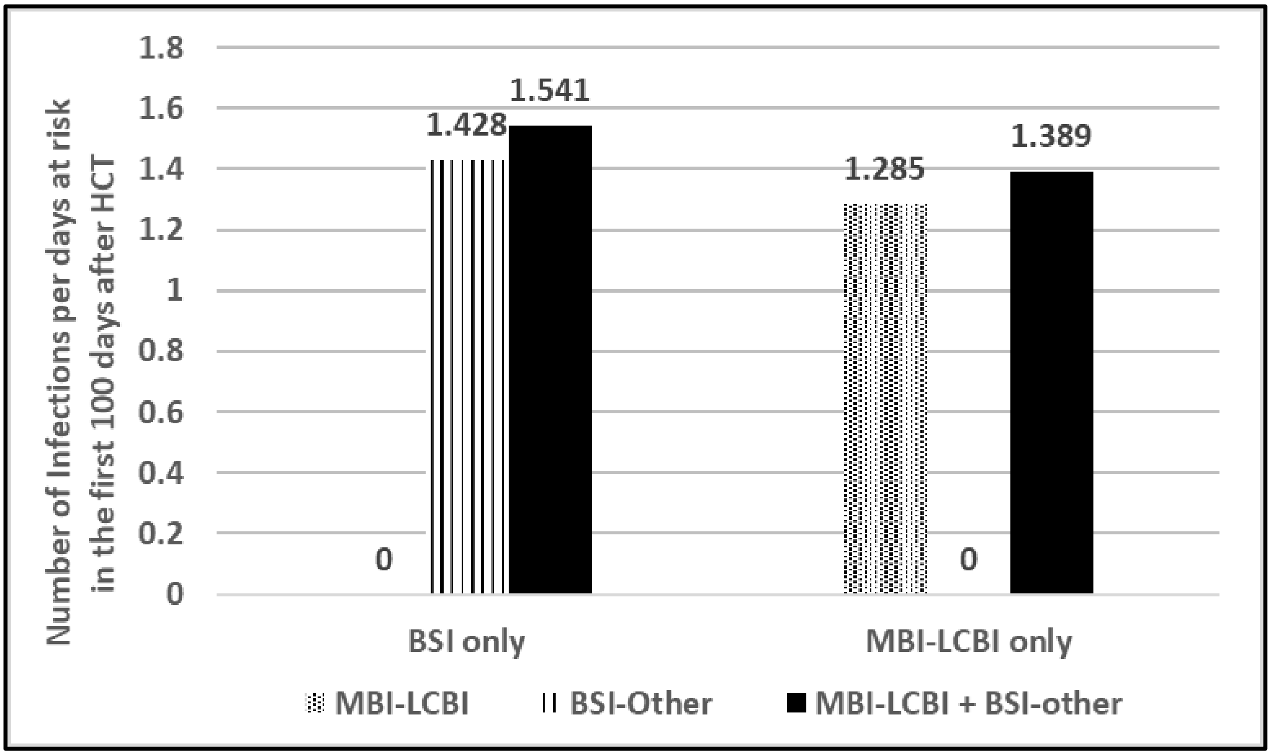

© 2020 Dandoy CE et al. *JAMA Network Open.*
